# 543. Sex Differences in Clinical Presentation and Outcomes of Adults Hospitalized with COVID-19 Pneumonia After Administration of Infliximab, Abatacept or Cenicriviroc: Subgroup Analysis from the ACTIV-1 Master Protocol

**DOI:** 10.1093/ofid/ofad500.612

**Published:** 2023-11-27

**Authors:** Adriana Rauseo, Tatyana Der, Radica Alicic, Bindu Balani, Caryn Morse, Pearl Zakroysky, Linda Shaw, Jane Atkinson, William G Powderly

**Affiliations:** Washington University School of Medicine in St Louis, St Louis, Missouri; Duke University School of Medicine, Durham, North Carolina; Providence Medical Research Center, Spokane, Washington; Hackensack University Medical Center, Hackensack, New Jersey; Wake Forest Baptist Hospital, Winston-Salem, North Carolina; Duke Clinical Research Institute, Durham, North Carolina; Duke Clinical Research Institute, Durham, North Carolina; National Center for Advancing Translational Sciences, Bethesda, Maryland; Washington University School of Medicine, St. Louis, Missouri

## Abstract

**Background:**

A growing body of evidence suggests potential sex differences in the clinical outcomes of COVID-19. In this study we evaluated whether sex and baseline clinical variables were associated with treatment effect after receipt of immunomodulators vs placebo in a study population derived from ACTIV-1 IM clinical trial.

**Methods:**

ACTIV-1 IM is a master protocol that evaluated infliximab, abatacept and cenicriviroc in addition to standard of care in adult patients hospitalized with moderate or severe SARS-CoV-2 infection through randomized, double-blind comparisons to shared placebo. We performed a subgroup analysis of the modified intention-to-treat cohort. Primary outcome was association of sex with time-to-recovery at day 28, key secondary outcomes were 28-day mortality and clinical status at day 14.

**Results:**

A total of 1381 participants were included. There was no difference in treatment effect for primary endpoint of time-to-recovery based on sex for infliximab (RRR 1.14, 95% CI 0.95-1.37 for males and RRR 1.09, 95% CI 0.90-1.34 for females with interaction p=0.77), abatacept (RRR 1.14, 95% CI 0.95–1.37 for males and RRR 1.13, 95% CI 0.91-1.40 for females with interaction p=0.93) or cenicriviroc (RRR 0.99, 95% CI 0.79–1.24 for males, RRR 0.98, 95% CI 0.77-1.24 for females, interaction p=0.93) substudies. There was no difference in treatment effect by sex for secondary outcomes for infliximab and abatacept substudies. The cenicriviroc substudy was terminated early for futility and found no difference in treatment effects based on sex for secondary outcomes either.

**Figure:**
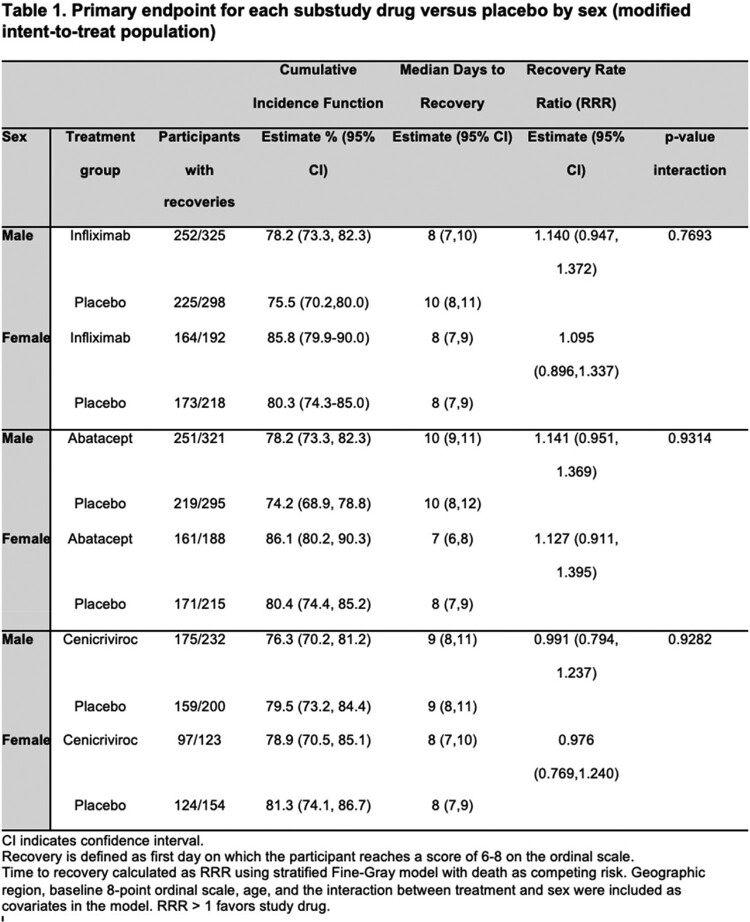

**Figure:**
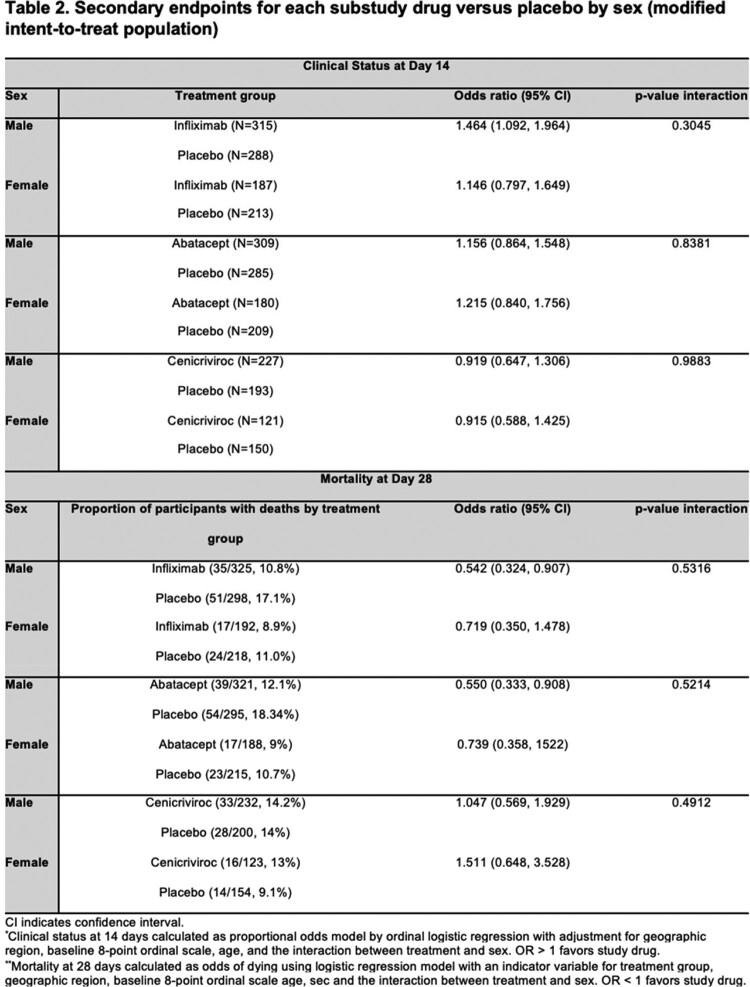

**Conclusion:**

There was no difference in treatment effect for infliximab, abatacept or cenicriviroc among males vs females. This suggests a more complex interplay of pathogen and host factors deserving further research to identify therapeutic targets for COVID-19.

**Disclosures:**

**Radica Alicic, MD, MSc**, Boehringer Ingelheim Pharmaceuticals, Inc.: Advisor/Consultant **Caryn Morse, MD**, Gilead Sciences Inc.: Grant/Research Support|Janssen: Grant/Research Support|Laurent Pharma: Grant/Research Support|Moderna: Grant/Research Support|Ridgeback Bio: Grant/Research Support **William G. Powderly, MD**, Merck: Advisor/Consultant

